# P-764. Defining Stewardship Opportunities Among Hospitalized Patients Prescribed Antibiotics for Positive Urine Cultures

**DOI:** 10.1093/ofid/ofaf695.975

**Published:** 2026-01-11

**Authors:** Alexander M Cyganowski, Rachel Marini, Ryan K Shields

**Affiliations:** UPMC, Pittsburgh, Pennsylvania; UPMC, Pittsburgh, Pennsylvania; University of Pittsburgh, Pittsburgh, PA

## Abstract

**Background:**

Urinary tract infections (UTI) are among a common indication for antibiotics in the hospital setting. Despite this, the diagnosis and management remain ill-defined leading to antibiotic overuse and challenges for stewardship programs. Our objective was to develop a targeted stewardship intervention for UTI.

**Figure:**
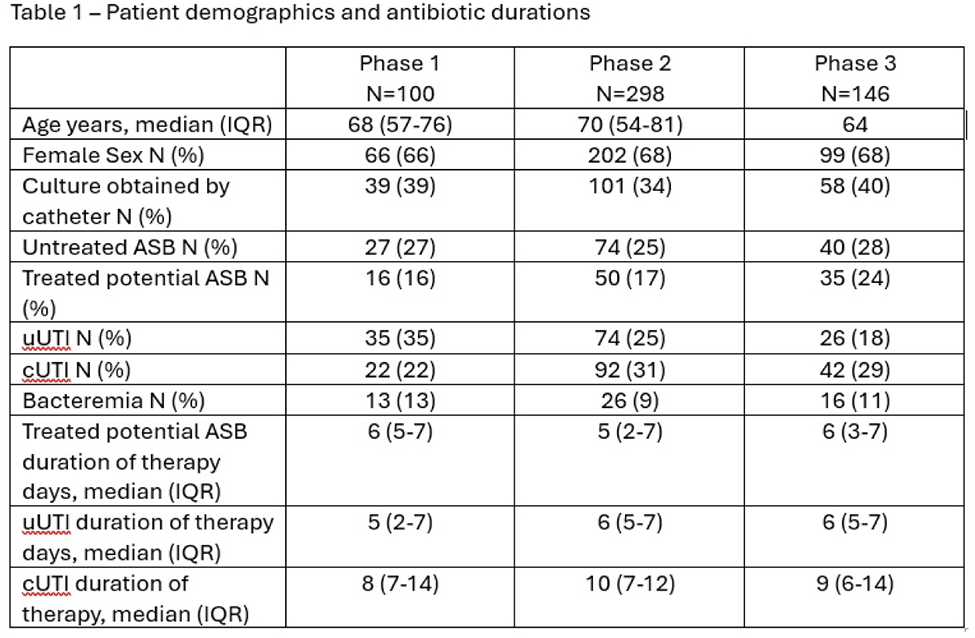

**Figure:**
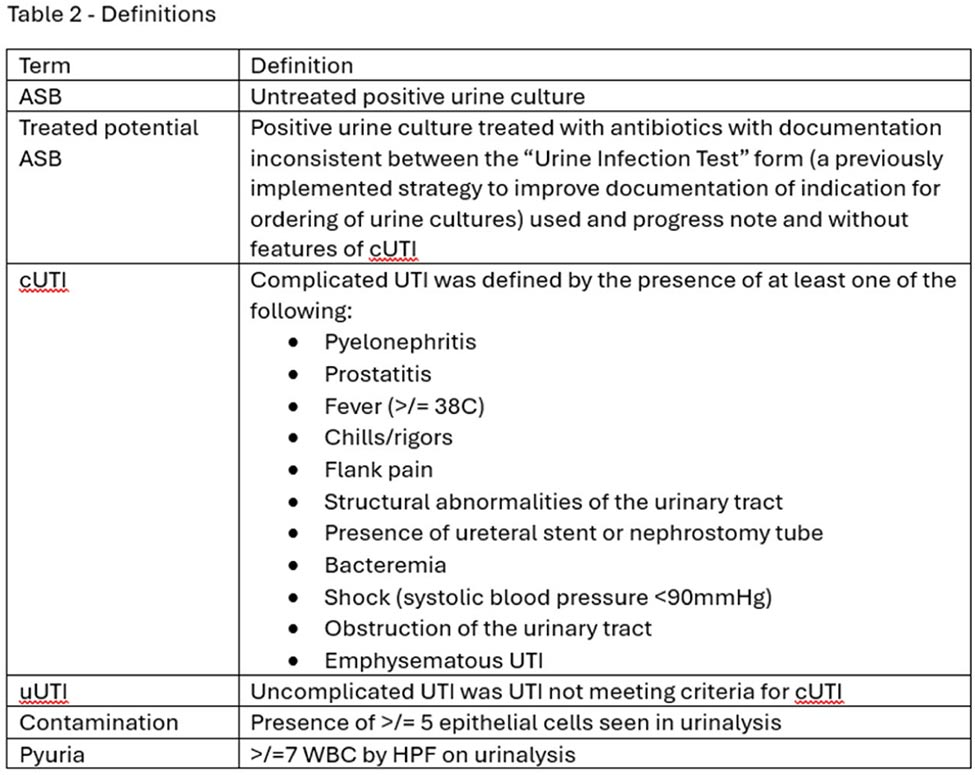

**Methods:**

Urinary tract infections (UTI) are among a common indication for antibiotics in the hospital setting. Despite this, the diagnosis and management remain ill-defined leading to antibiotic overuse and challenges for stewardship programs. Our objective was to develop a targeted stewardship intervention for UTI.

**Figure:**
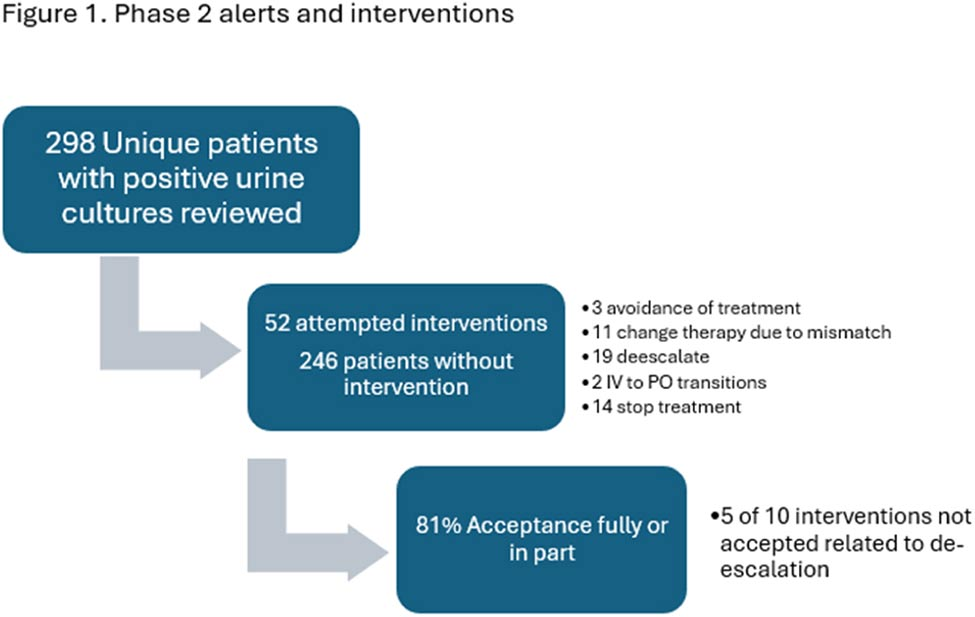

**Figure:**
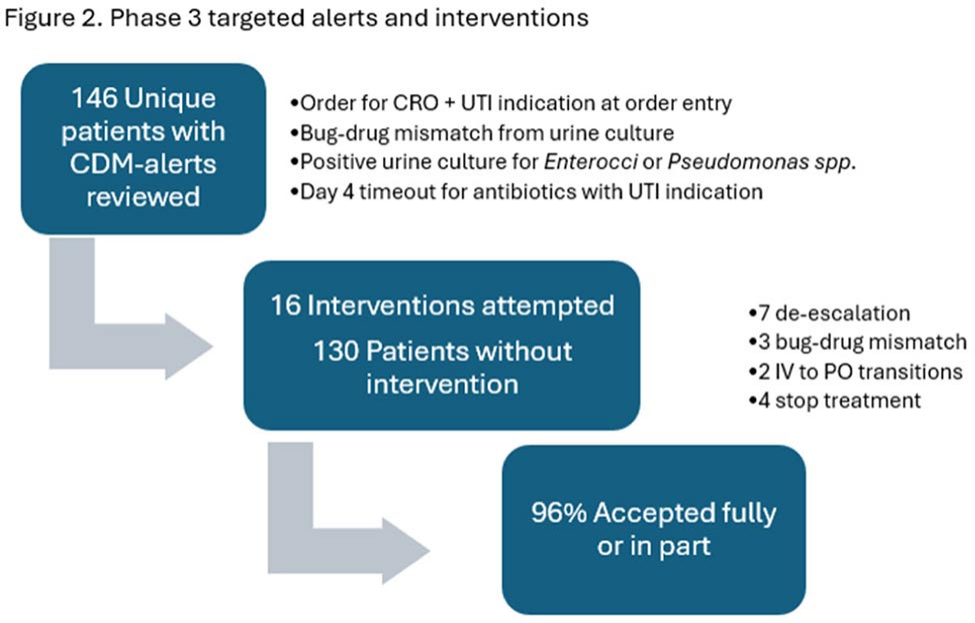

**Results:**

A total of 544 patients were included in the study (Table 1). In phase 1, we found that 27%, 16%, 35%, and 22% of positive urine cultures were associated with asymptomatic bacteriuria (ASB), treated potential ASB, uncomplicated UTI (uUTI), and complicated UTI (cUTI), respectively (Table 2); 22 samples were contaminated. Empiric antibiotics were in vitro active in 73% of cases overall, including 91% and 63% of cUTI and treated ASB/uUTI, respectively. 77% of patients with cUTI received an anti-pseudomonal beta-lactam empirically, that was de-escalated in 63% (14/22). In phase 2, an alert notified the stewardship team of each positive urine culture for 293 consecutive patients, but only 17% resulted in potential interventions (Figure 1). In phase 3, the alert was modified with CDM to include only high priority alerts, including bug-dug mismatch, long durations of treatment, or unnecessarily broad treatment. During this phase, the number of alters was reduced significantly and the rate of accepted interventions increased to 96% (Figure 2).

**Conclusion:**

A key finding from our broad approach to UTI was that a significant proportion of positive urine cultures were never treated or contaminated, suggesting that urine is over-cultured among hospitalized patients. Among patients with UTI requiring treatment, our stepwise approach reduced the number of alerts to those that were actionable for our stewardship team providing a framework for broader implementation.

**Disclosures:**

All Authors: No reported disclosures

